# Clinician Perspectives on Workflow, EHR Integration, and Dementia Care Management for Hospice Transitions

**DOI:** 10.1093/geroni/igaf122.3896

**Published:** 2025-12-31

**Authors:** Daniella Torres, Komal Murali

**Affiliations:** NYU College of Global Public Health, New York, New York, United States; New York University Rory Meyers College of Nursing, New York, New York, United States

## Abstract

This study explores healthcare professional perspectives on workflow, electronic health record (EHR) use, and care management during hospice transitions for persons with dementia and their care partners. Eighteen nurses, nurse practitioners, and physicians (with experience in acute care and home healthcare) participated across three semi-structured virtual focus groups. Inductive coding was applied to identify key themes. The thematic analysis revealed three interconnected domains of facilitating optimal hospice transitions: structured workflows, EHR integration, and optimizing care manager roles. Participants emphasized the importance of structured, multi-step communication to clarify responsibilities, improve care partner support, and ensure continuity of care. Workflow integration was described as requiring clinical flexibility to balance thoroughness with feasibility, including proper timing, patient privacy, follow-up, and interdisciplinary collaboration. EHR use emerged as central to supporting seamless workflow and documentation. Clinicians preferred direct messaging over automated flags to reduce alert fatigue and favored embedding hospice transitions checklists and advanced care planning templates within the EHR to facilitate information sharing and linking documentation to actionable outcomes. Care managers were identified as facilitators rather than decision-makers who provide targeted guidance and have defined responsibilities in hospice transitions. Telephonic, virtual, and in-person communication were highlighted as context-dependent strategies, complemented by follow-up hospice resources for caregivers. Findings suggest that hospice transitions care relies on structured workflows, EHR optimization, and care manager roles. Interventions that balance standardized tools with personalized communication and provider workflow and caregiver experience may improve the quality and consistency of hospice care delivery for persons with dementia and their care partners.

